# Effect of Different Pressures of Supercritical Carbon Dioxide on the Microstructure of PAN Fibers during the Hot-Drawing Process

**DOI:** 10.3390/polym11030403

**Published:** 2019-03-01

**Authors:** Mengmeng Qiao, Haijuan Kong, Xiaoma Ding, Zhifeng Hu, Luwei Zhang, Yuanzhi Cao, Muhuo Yu

**Affiliations:** 1 State Key Laboratory for Modification of Chemical Fibers and Polymer Materials, College of Materials Science and Engineering, Donghua University, Shanghai 201620, China; 2160320@mail.dhu.edu.cn (M.Q.); 1159124@mail.dhu.edu.cn (X.D.); 2160226@mail.edu.cn (Z.H.); 2170293@mail.dhu.edu.cn (L.Z.); 2180410@mail.dhu.edu.cn (Y.C.); 2 School of Materials Engineer, Shanghai University of Engineer Science, Shanghai 201620, China

**Keywords:** hot-drawing, polyacrylonitrile fibers, supercritical carbon dioxide, crystallinity, degree of orientation, mechanical property

## Abstract

The hot-drawing process of polyacrylonitrile (PAN) fibers is an important step during the production of PAN-based carbon fibers. In this study, supercritical carbon dioxide (Sc-CO_2_) was used as one kind of media for thermal stretching of PAN fibers to study the effect of different pressures of Sc-CO_2_ on crystallinity, degree of orientation and mechanical property of PAN fibers during the hot-drawing process. The changes of microstructure and mechanical properties in the PAN fibers were investigated by wide-angle X-ray diffraction, small angle X-ray scattering and monofilament strength analysis. The results showed that as the pressure increased, the crystallinity and degree of orientation of PAN fibers increased. Furthermore, when the pressure was 10 MPa, the crystallinity increased from 69.78% to 79.99%, which was the maximum crystallinity among the different pressures. However, when the pressure was further increased, the crystallinity and degree of orientation of the fibers were reduced. The test results of the mechanical properties were consistent with the trends of crystallinity and degree of orientation, showing that when the pressure was 10 MPa, the tensile strength of the fibers increased from 4.59 cN·dtex^−1^ to 7.06 cN·dtex^−1^ and the modulus increased from 101.54 cN·dtex^−1^ to 129.55 cN·dtex^−1^.

## 1. Introduction

Carbon fibers (CFs) are defined as one kind of high performance fiber containing at least 92 wt % carbon [1] which have achieved global interest owing to the advantages of high tensile properties, low densities, high thermal and chemical stabilities. Because of these superior properties, CFs are well known as carbonaceous reinforcing materials utilized increasingly in fields ranging from the aerospace industry to sport products [2,3,4]. CFs can be produced from many different precursors, such as polyacrylonitrile (PAN) [5], mesophase pitch [6,7], rayon, etc. [8], among which PAN-based CFs are the preferred reinforcements for structural composites owing to their perfect strength, stiffness combined with their light weight as well as lower cost. Therefore, the majority of CFs (about 95%) are made from PAN fibers [9]. PAN is one kind of polymer with the chain of carbon connected to one another which is hard, horny, relatively insoluble, and has high-melting material [10]. According to the continuity of polymerization and spinning, it is generally divided into one-step and two-step methods. According to the spinning process, it is generally divided into wet spinning [11], dry spinning [12], dry-jet wet spinning [13] and melting spinning, among which dry-formed fiber structure is the tightest. The change of fineness of fibers in wet forming is small, the solvent remaining in the fibers is small, and the quality of the raw silk can be easily controlled, which results in wet forming being widely used in the spinning process. The spinning solvents are dimethyl sulfide [14], dimethyl-formamide [15], sodium thiocyanate [16], zinc chloride [17], nitric acid, etc.

At present, experts and scholars at home and abroad all believe that the quality of PAN precursors is key in determining the performance of CFs, that is to say, only high-performance PAN precursors can produce high-performance CFs. Therefore, we can study new ways to improve the performance of PAN fibers, thereby improving the performance of CFs.

Pure CO_2_ has a low critical temperature and pressure (*T*_c_ = 304.1 K and *P*_c_ = 73.7 bar), which can be used in moderate process conditions. Supercritical carbon dioxide (Sc-CO_2_) is usually used as non-toxic solvents in extractions [18,19], separations [20,21], chemical reactions [22], and various other applications [23,24,25,26]. At present, more and more experts use Sc-CO_2_ to induce the crystallization of polymers and fibers [27,28,29]. Owing to the characteristics of the Sc-CO_2_ fluid, it can be applied to the hot-drawing process of PAN fibers at low temperatures. In this paper, we tried to find a new way to prepare the PAN-based CFs with higher performance, and Sc-CO_2_ was used to treat the PAN fibers during the hot-drawing process. Due to the permeability and plasticization of Sc-CO_2_, CO_2_ entered the interior of the fibers which may have induced crystallization of PAN fibers under the combined effect of force and heat. Furthermore, the crystallinity and degree of orientation of the fibers increased under the action of force and heat, thereby increasing the mechanical properties of the fibers.

## 2. Experiments

### 2.1. Materials and Sample Preparation 

PAN fibers (having 24,000 filaments per bundle) were supplied by Weihai Fiber Development Co., Ltd. (Weihai, China). CO_2_ with the purity of 99.99% was supplied by Sinopharm Chemical Reagent Co., Ltd. (Suzhou, China).

**Figure 1:**
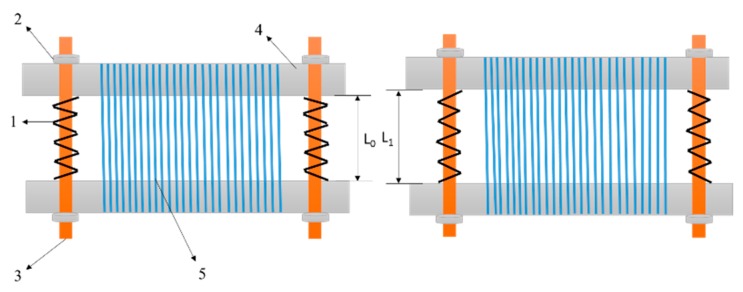
Tension application instrument: (1) Spring; (2) nut; (3) screw; (4) semicircular stainless-steel tube; (5) polyacrylonitrile (PAN) fibers; *L*_0_: Spring length after loosening the nut; *L*_1_: Spring length after reaction.

First, we prepared a tension application instrument which is shown in Figure 1. Secondly, we compressed the spring to a certain length, fixed it with a nut, and then wrapped the PAN fibers with 4 turns. After the fibers were fixed, the nut of the fixing spring was unscrewed and then the tension application instrument was placed in the reaction vessel when the high-pressure and high-temperature reactor was heated to a certain temperature. We opened the intake valve and the outlet valve of the reactor and removed the air in the reactor with a small amount of CO_2_ gas three times, and then pressured it to a certain pressure with the CO_2_ we required. After a period of reaction, the CO_2_ gas was discharged, and the hot-drawing PAN fibers were obtained. 

### 2.2. Characterization

The stress-strain curve of a single fiber was recorded using a XQ-2 tensile tester with a gauge length of 20 mm and an extension rate of 20 mm·min^−1^. At least 50 fibers were tested for each sample and the average tensile strength, elongation at break and initial modulus were calculated.

Small angle X-ray scattering (SAXS) analysis of the fibers was carried out at the Shanghai Synchrotron Radiation Facility (SSRF) on beam line (BL14B) with an X-ray wavelength of 0.124 nm. The sample-to-detector (Mar CCD 165) distance was 1980 mm. All data analysis was carried out using the X-polar software (Precision Works NY, Inc., Schenectady, NY, USA).

Wide angle X-ray diffraction (WAXD) was carried out at the Shanghai Synchrotron Radiation Facility (SSRF) on beam line (BL14B) with an X-ray wavelength of 0.124 nm. A bundle of PAN fibers was placed on a sample holder with the fiber direction perpendicular to the X-ray beam. The specimen-to-detector (Mar 345) distance was calibrated using the standard sample LaB6. All data analysis (background correction, radial and azimuthal integration) was carried out using X-polar software (Precision Works NY, Inc., Schenectady, New York, USA). The degree of crystallinity of the fiber was calculated using Equation (1):

(1) $$W_{c}=\frac{l_{c}}{l_{c}+l_{a}}\times 100\%$$

where *W*_c_ is the crystallinity of the fiber, and *l*_c_ and *l*_a_ are the total peak area of the crystal phase and amorphous phase, respectively.

## 3. Results and Discussion

### 3.1. Mechanical Properties of PAN Fibers Treated at Different Pressures

**Figure 2:**
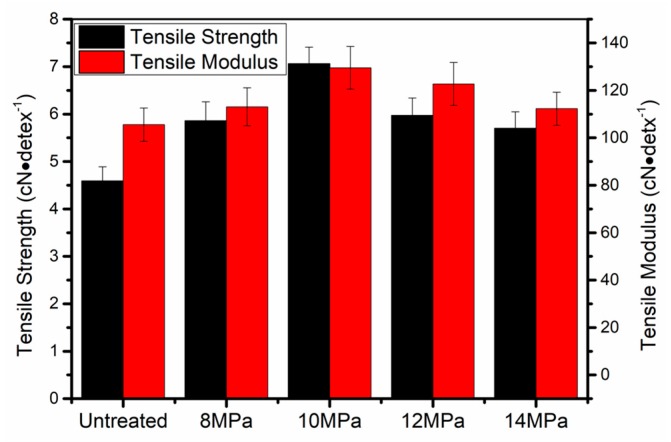
Mechanical performance of PAN fibers treated at different pressures in supercritical carbon dioxide (Sc-CO_2_).

The mechanical properties of the PAN fibers treated in Sc-CO_2_ at different pressures were tested in this article and the changes in the mechanical properties of PAN fibers were obtained, as shown in Figure 2. Compared with the untreated PAN fibers, the tensile strength of the PAN fibers increased from 4.59 cN·dtex^−1^ to 7.06 cN·dtex^−1^ when treated pressure was 10 MPa. In addition, it was of high interest to note that the change in pressures were very small. That is to say, the tensile strength of the PAN fibers was very sensitive to pressure which might be attributed to changes in CO_2_ concentration. The tensile modulus of PAN fibers treated at different pressures are shown in Figure 2, from which it is not hard to observe that the variation trend of Young’s modulus was similar to tensile strength. Under the action of tension and heat, the molecular chains of PAN fibers moved in the direction of tension, which increased the degree of orientation of the fibers. Owing to the movement of PAN chains and the effect of tension, the defects in the fibers were reduced. The crystallinity and degree of orientation of the fibers were used to determine the mechanical properties of the fibers in a certain aspect. Therefore, the change trend of mechanical properties was the same as the trend of crystallinity and degree of orientation. The experiment showed that the PAN fibers with the best mechanical properties could be obtained after hot-drawing at 10 MPa.

### 3.2. Wide Angle X-Ray Diffraction (WAXD) Analysis of PAN Fibers Treated at Different Pressures

**Figure 3:**
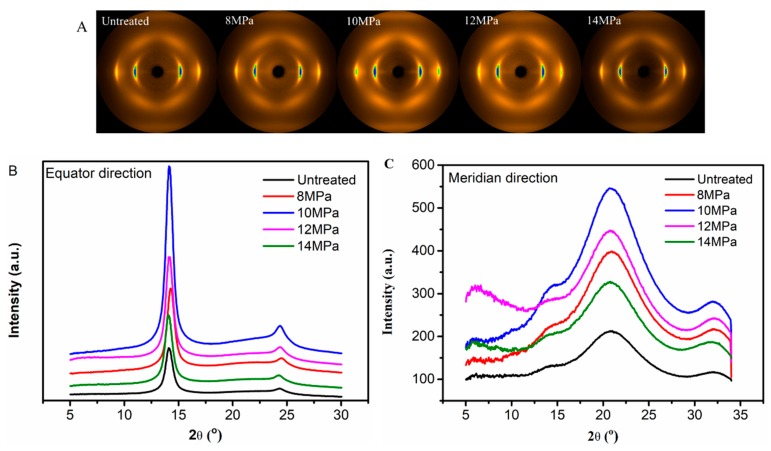
Wide angle X-ray diffraction (WAXD) patterns of PAN fibers treated with different pressures: (**A**) 2D-WAXD diffraction patterns; (**B**) integral diffraction intensity of equatorial line; (**C**) meridian integral diffraction intensity.

Crystal structure and molecular orientation played important roles in the properties of PAN fibers. The 2D-WAXD patterns of PAN fibers treated at different pressures are given in Figure 3A, from which we can observe that the PAN fibers had two distinct diffraction fringes in the equatorial direction, the crystal planes of (100) and (110), respectively. At the same time, there were four diffraction arcs in the meridional direction, which were symmetrically distributed along the central axis. As the pressure increased, the diffraction fringes in the equatorial direction became shorter and sharper, indicating an increase in crystallinity and the degree of orientation. However, as the pressure increased further, the diffraction fringes in the equatorial direction gradually became longer and weaker. 

**Figure 4:**
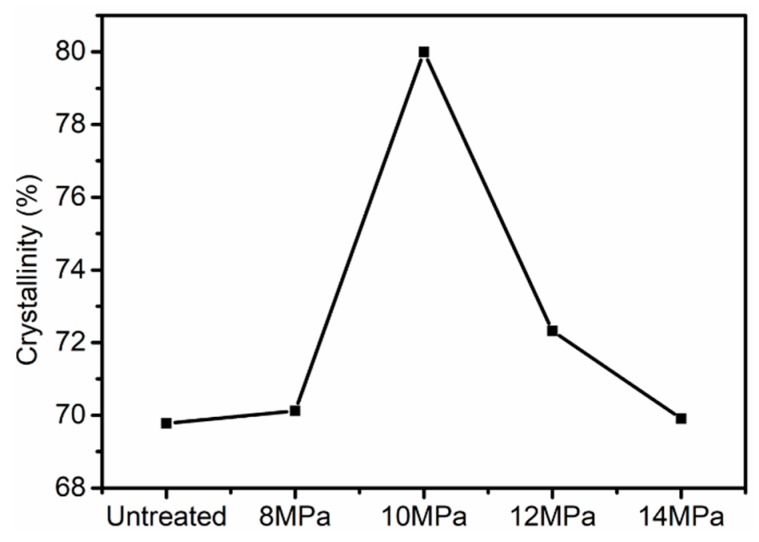
Crystallinity of PAN fibers treated at different pressures in Sc-CO_2_.

The equatorial and meridional radial integrations of 2D-WAXD patterns of PAN fibers treated at various pressures are shown in Figure 3B,C respectively. The radial intensities in equatorial direction were obtained by integrating over −15° ≤ φ ≤ 15°, where φ denoted the azimuth angle. From Figure 3B, it can clearly be seen that two peaks appeared at 2θ = 13.6° and 2θ = 23.6° in integral spectrum of equatorial direction, reflecting the ordered crystal structure in PAN fibers. The stronger diffraction peak at 2θ = 13.6° corresponding to the crystal plane of (100), reflected the molecular chain spacing. In addition, the weaker diffraction peak at 2θ = 23.6° corresponding to the crystal plane of (110), reflected the distance between nearly parallel molecular pieces. As the pressure increased, the concentration of CO_2_ and the force in the reactor increased gradually, allowing us to also analyze the effects of the concentration of CO_2_ and force on the crystallization behavior of PAN fibers. The intensities of (100) and (110) of the treated fibers were increased with the increase of pressures at first, indicating that crystallization was increased with the increase of CO_2_ concentration. With the further increase of pressure, the intensities of (100) and (110) planes were decreased, indicating that crystallinity would decrease if the concentration of CO_2_ was too high. The PAN fibers were plasticized and swelled due to the presence of Sc-CO_2_ so that the movement of molecular chains was enhanced, and the molecular chains were rearranged under the tension just like Figure 7, causing an increase in crystallinity and degree of orientation. As data on crystallinity of the PAN fibers show in Figure 4, when the pressure was 10 MPa, the crystallinity increased from 69.78% to 79.99%, however, the crystallinity was only 69.91% when the pressure was 14 MPa. We explained these changes using the following aspects: When the Sc-CO_2_ was dissolved in the PAN fibers, the free volume of the fibers increased, and the force between the segments was weakened which was good for the movement of molecular chains. At the same time, the flexibility of the segments was increased, which allowed the fibers to crystallize below the crystallization temperature. However, when the system pressure was too high, hydrostatic pressure was generated [30,31]. Therefore, the free volume of the PAN fibers shrank, the movement of the segment was restricted, and the difficulty of the segment movement was increased, which all resulted because the crystallinity of the PAN fibers was lower when the pressure was too high. We can conclude that the crystallinity of PAN fibers increased with the increase in pressure, and then showed a downward trend when the pressure was further increased.

The radial intensities in meridional direction were obtained by integrating over 15° ≤ φ ≤ 165° shown in Figure 3C, which showed that there were four broad scattering peaks of amorphous matrix of PAN fibers appeared at 2θ = 6.3°, 2θ = 13.6°, 2θ = 21.6° and 2θ = 32°, respectively. These four broad scattering peaks of amorphous matrix were sharper when the pressure was 10 MPa which indicated that after treated in the medium of Sc-CO_2_ under the presence of heat and tension, the PAN fibers had a higher degree of orientation. 

**Figure 5:**
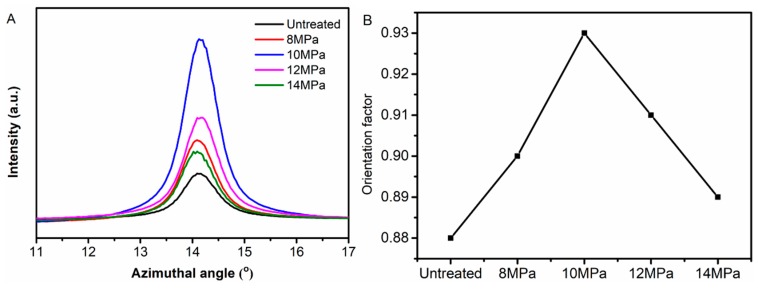
(**A**) Azimuthal scan curves of the (100) plane and (**B**) Orientation factor of (100) plane of PAN fibers treated at different pressures.

The orientation factor f_c_ was a good parameter to describe the orientation of crystal. The plane of (100) was often used for the calculation of crystal orientation in PAN fibers, and (002) for the analysis of graphite in CFs. The crystal orientation along the fiber axis was calculated based on the Herman’s orientation function as shown in Equation (2):

(2) $$f_{c}=\frac{3{\langle cos^{2}\varphi \rangle }_{100}-1}{2}$$

where ${\langle cos^{2}\varphi \rangle }_{100}$ was defined as:

(3) $${\langle cos^{2}\varphi \rangle }_{100}=\frac{\int_{0}^{\pi /2}I\left(\varphi_{100}\right)cos^{2}\varphi_{100}sin\varphi_{100}d\varphi_{100}}{\int_{0}^{\pi /2}I\left(\varphi_{100}\right)sin\varphi_{100}d\varphi_{100}}$$

where φ_100_ was the azimuthal angle, I(φ_100_) was the diffraction intensity distribution of the reciprocal lattice vector of the (100) plane. Figure 5A shows the azimuthal scan curves of the (100) plane and Figure 5B shows the orientation of the (100) plane of PAN fibers. We found that the orientation factor f_c_ showed a trend of increasing first and then decreasing with the increase of pressure.

We could explain the changes in the degree of orientation using the following aspects: When Sc-CO_2_ was dissolved in the PAN fibers, the segments of fibers moved in the direction of tension which increased the degree of orientation. However, when the system pressure was too high, hydrostatic pressure would be generated, the movement of the segment was restricted, and the difficulty of the segment movement was increased, which all led to the reduction in the degree of crystallization.

### 3.3. Small Angle X-Ray Scattering (SAXS) Analysis of PAN Fibers Treated at Different Pressures

**Figure 6:**
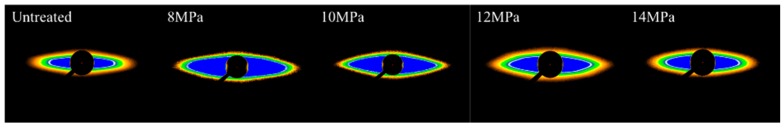
Small angle X-ray scattering (SAXS) patterns of PAN fibers treated at different pressures.

Small angle X-ray scattering (SAXS) technology is an effective method for studying the microstructure and microvoids in fibers due to the penetration of X-ray and its statistical results which can obtain structural features in the range of 1–100 nm length. For the PAN-based CFs, it is well known that the contribution of SAXS was attributed to the needle-shaped microvoids which had an orientation distribution parallel to the axis direction of the fibers [32,33,34]. Figure 6 shows the SAXS patterns of PAN fibers treated at different pressures during the hot-drawing process. Diamond shaped patterns appeared in the SAXS patterns, which was due to the rod-like structure oriented along the stretching direction. From the SAXS patterns, it can be clearly seen that the pattern of the untreated fibers exhibited long oval shapes. With the increase of pressure, the pattern gradually became sharper in the direction of the equator. However, the pattern gradually became more rounded in the direction of the equator with further increases in pressure.

The invariant *Q* gave information about the content of microvoids for isotropic scattering. The one-dimensional pseudo-invariant *Q*_ψ_ had a relationship with the orientation of microvoids for anisotropic scattering, which is defined in Equation (4) [35]:

(4) $$Q_{\psi }=\int_{0}^{+\infty }q^{2}I_{\psi }\left(q\right)dq$$

where ψ was the angle between the direction of fiber axis and the direction of integral-strip; *q* was the scattering vector (*q* = 4πsinθ/λ, λ was the wavelength and 2θ was the scattering angle). 

The parameter *L*_2_ characterized the microvoids and was calculated based on the method of Shioya and Takaku [33,36] as shown in Equation (5):

(5) $$L_{2}=\frac{2\int_{0}^{+\infty }I\left(\vec{q}\right)dq_{3}dq_{12}}{Q}$$

where *L*_2_ was the average chord length of a line drawn through the cross-section of the voids at random; *q*_12_ and *q*_3_ were the components of the reciprocal space vectors in the horizontal and vertical direction, respectively. 

The orientation distribution *B*_ψ_ and the micropore length L of the voids along with the fiber axis direction were obtained by the method of Ruland [34]. For the PAN fibers, the azimuthal scans of the equatorial streak were conducted in accordance with the Lorentzian function, which was used to estimate the observed integral breadth B_obs_. The integral breadth *B*_obs_ had a relationship with *q*, at the same time, for different values of q, the microvoid length (*L*) and the misorientation width (*B*_ψ_) were calculated from the Cauchy–Cauchy formula as Equation (6):

(6) $$B_{obs}=B_{\psi }+\frac{2\pi }{Lq}$$

where *B*_obs_ was the full width at the half maximum of the azimuthal profile from the equatorial streak.

The micropore length L and orientation deviation angle *B*_ψ_ in the fibers were calculated and are shown in Table 1. From Table 1, we observe that with the increase of pressure, the micropore length reduced, which indicated that as the concentration of CO_2_ increased, the movement of the segments led to a reduction of hollow space in the fibers. At the same time, information about the changes of micropore’ orientation was obtained from data on *B*_ψ_, which showed that with the increase in pressure, *B*_ψ_ reduced, while with the further increase of pressure, *B*_ψ_ increased. The possibilities surrounding this phenomenon might be explained by two aspects: First, as pressure increased, the force received by the fiber gradually increased, which might have caused the micropores to change along the direction of fiber axis, resulting in the decrease of *B*_ψ_. On the other hand, under the action of Sc-CO_2_, the molecular chains in the fibers moved easily along the axis of the fibers, which might have caused the orientation of micropores to increase in the same direction. While with the further increase of pressure, the movement of molecular chains in the fibers were hindered because of the hydrostatic pressure generated by CO_2_ and thus, *B*_ψ_ began to reduce. However, *B*_ψ_ increased slightly faster than the untreated fibers which indicated that the movement of molecular chains played a more important role than the pressure force generated by pressure.

### 3.4. Stretching Behavior Analysis of the PAN Fibers Heated at Different Pressures

**Figure 7:**
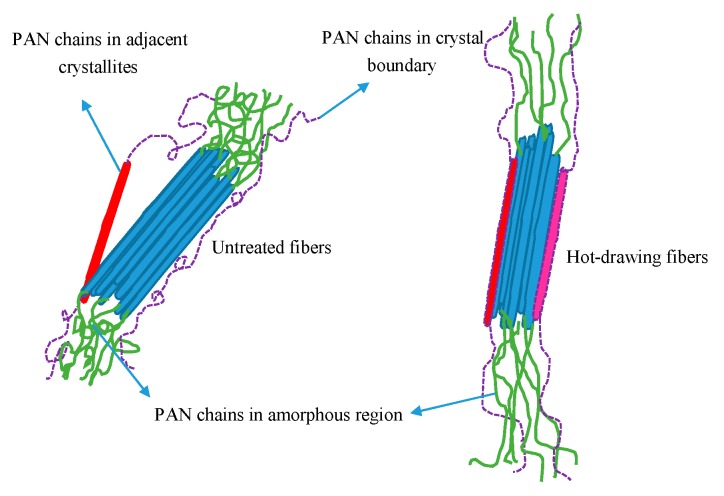
Schematic diagram of the change of crystallinity and the variation of chain segment in amorphous zone of fibers.

**Figure 8:**
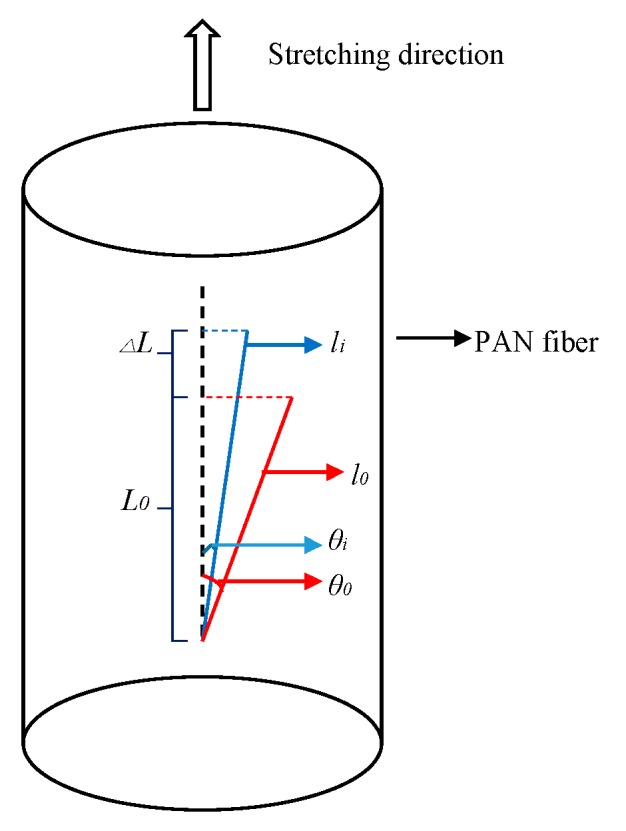
Schematic diagram of the deformation behavior of the PAN chain during hot stretching.

The glass transition temperature (*T*_g_) of PAN fibers was 100 °C. When the temperature was above *T*_g_, thermal motion occurred among the molecular chains. Owing to the presence of heat and tension, the molecular segments in the amorphous region moved along the direction of the tension which resulted in molecular chains oriented in the direction of fiber axis. As shown in Figure 7, the molecular segments in the amorphous region underwent thermal motion under the action of heat and tension in Sc-CO_2_ fluid and the molecular chains were oriented along the axial direction, so that the molecular segments of the amorphous regions in the fibers were more regularly arranged which increased the degree of orientation of the fibers. At the same time, there were some PAN chains where crystal boundary crystallized after the hot-drawing process, as shown as Figure 7. It was well known that the length and diameter of the fibers would change after hot-drawing process, as shown in Figure 8. Owing to the molecular chains of the fibers oriented along the axial direction of the fiber, the angle between the fiber segments and the fiber axis reduced, which resulted in the length of the fiber increasing. As shown in Figure 8, the chain length of PAN fibers in the quasi-crystal was *l*_0_, and the dislocation angle relative to the fiber axis was θ_0_. When the fiber was stretched under a certain tension, the length of the PAN chain increased to l_i_ along the fiber axis due to tension in the direction of the fiber axis. The change in the length of PAN fibers could be calculated from the length of the spring before and after the reaction. Therefore, we calculated the elongation of the PAN fibers after hot-drawing, according to Equation (7):

(7) $$E=\;\frac{L-L_{0}}{L_{0}}\times 100\%$$

where *L*_0_ was the length of the spring after loosening the nut; *L* was the spring length after reaction.

**Figure 9:**
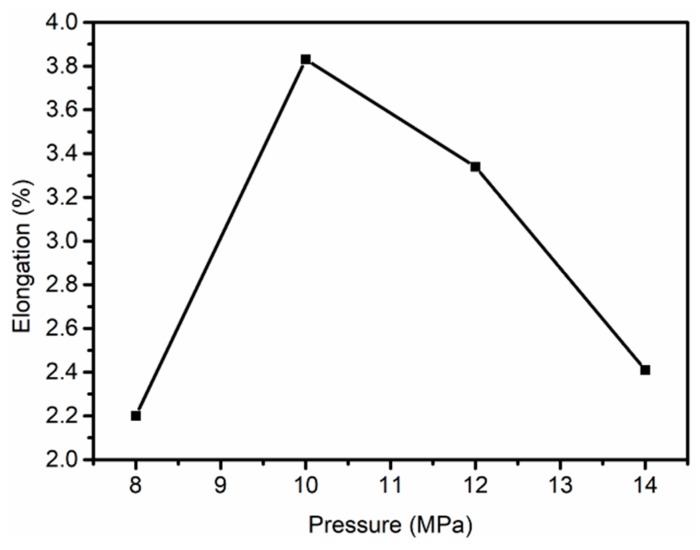
Elongation of PAN fibers at different pressures in Sc-CO_2_.

It can be seen in Figure 9 that the maximum elongation of the PAN fibers in the Sc-CO_2_ fluid was 3.38%, which indicates that the Sc-CO_2_ fluid plasticized the fibers during the thermal stretching process, indicating that Sc-CO_2_ played an important role in the PAN fibers during the hot-drawing process.

## 4. Conclusions

In this study, the experiment showed that when the PAN fibers were treated at 10 MPa in Sc-CO_2_, the tensile strength of the PAN fibers increased from 4.59 cN·dtex^−1^ to 7.06 cN·dtex^−1^, the modulus of PAN fibers increased from 101.54 cN·dtex^−1^ to 129.55 cN·dtex^−1^. The crystallization data calculated by wide angle X-ray diffraction showed that when the PAN fibers were treated at 10 MPa in Sc-CO_2_, the crystallization increased from 69.78% to 79.99%. These findings all indicated that it was a useful method for preparing PAN fibers with high strength.

## Figures and Tables

**Table 1:** The characterization of microvoid structures in PAN fibers at different pressures during the hot-drawing process.

Sample	*B*_ψ_ (^o^)	L (nm)
Untreated	28.27777	1626.741
8 MPa	24.35159	340.1361
10 MPa	17.95414	163.6661
12 MPa	18.03325	187.2659
14 MPa	27.73146	373.1343

## References

[1] Park S.-J. (2018). History and Structure of Carbon Fibers.

[2] Park S.-J. (2018). Novel Carbon Fibers and Their Composites.

[3] Zhi-Ping G.U. (2017). Development and application of carbon fiber brush for DC motor. Carbon.

[4] Barile C., Casavola C. (2018). Mechanical characterization of carbon fiber reinforced plastics specimens for aerospace applications. Polym. Compos..

[5] Kim S., Kuk Y.-S., Chung Y.S., Jin F.-L., Park S.-J. (2014). Preparation and characterization of polyacrylonitrile-based carbon fiber papers. J. Ind. Eng. Chem..

[6] Shin S., Jang J., Yoon S.H., Mochida I. (1997). A study on the effect of heat treatment on functional groups of pitch based activated carbon fiber using FTIR. Carbon.

[7] Yao Y., Chen J., Ling L., Dong Y., Liu A. (2014). Mesophase pitch-based carbon fiber spinning through a filter assembly and the microstructure evolution mechanism. J. Mater. Sci..

[8] Zhang X.Y., Amp S. (2017). Technical Advances and Development Suggestions for Rayon-based Carbon Fiber and Pitch-based Carbon Fiber. Chem. Fertil. Des..

[9] Frank E., Hermanutz F. (2012). Carbon Fibers: Precursors, Manufacturing, and Properties. Macromol. Mater. Eng..

[10] Clauser H.R. (1976). Encyclopedia/Handbook of Materials, Parts, and Finishes.

[11] Zeng X., Hu J., Zhao J., Zhang Y., Pan D. (2007). Investigating the jet stretch in the wet spinning of PAN fiber. J. Appl. Polym. Sci..

[12] Ohzawa Y., Nagano Y. (1970). Studies on dry spinning. II. Numerical solutions for some polymer–solvent systems based on the assumption that drying is controlled by boundary-layer mass transfer. J. Appl. Polym. Sci..

[13] Wang T.-Y., Chang H.-C., Chiu Y.-T., Tsai J.-L. (2014). The index of dry-jet wet spinning for polyacrylonitrile precursor fibers. J. Appl. Polym. Sci..

[14] Dong X.-G., Wang C.-G., Bai Y.-J., Cao W.-W. (2007). Effect of DMSO/H_2_O coagulation bath on the structure and property of polyacrylonitrile fibers during wet-spinning. J. Appl. Polym. Sci..

[15] Chen J., Harrison I. (2002). Modification of polyacrylonitrile (PAN) carbon fiber precursor via post-spinning plasticization and stretching in dimethyl formamide (DMF). Carbon.

[16] Xu L., Qiu F. (2015). Unusual viscosity behavior of polyacrylonitrile in NaSCN aqueous solutions. Polymer.

[17] Cho S.H., Park J.S., Jo S.M., Chung I.J. (1994). Influence of ZnCl_2_ on the structure and mechanical properties of polyacrylonitrile fibers. Polym. Int..

[18] Friedrich J.P., Pryde E.H. (1984). Supercritical CO_2_ extraction of lipid-bearing materials and characterization of the products. J. Am. Oil Chem. Soc..

[19] Baldino L., Della Porta G., Reverchon E. (2017). Supercritical CO_2_ processing strategies for pyrethrins selective extraction. J. CO2 Util..

[20] Bonthuys G.J.K., Schwarz C.E., Burger A.J., Knoetze J.H. (2011). Separation of alkanes and alcohols with supercritical fluids. Part I: Phase equilibria and viability study. J. Supercrit. Fluids.

[21] Bonthuys G.J.K., Schwarz C.E., Burger A.J., Knoetze J.H. (2008). Separation of alkanes and alcohols with supercritical fluids. J. Supercrit. Fluids..

[22] Clifford A.A. (1994). Reactions in Supercritical Fluids.

[23] Arranz E., Jaime L., Hazas M.C.L.D.L., Reglero G., Santoyo S. (2015). Supercritical fluid extraction as an alternative process to obtain essential oils with anti-inflammatory properties from marjoram and sweet basil. Ind. Crop. Prod..

[24] Elgndi M.A., Filip S., Pavlić B., Vladić J., Stanojković T., Žižak Ž., Zeković Z. (2017). Antioxidative and cytotoxic activity of essential oils and extracts of Satureja montana L., Coriandrum sativum L. and Ocimum basilicum L. obtained by supercritical fluid extraction. J. Supercrit. Fluids.

[25] Baldino L., Reverchon E. (2017). Challenges in the production of pharmaceutical and food related compounds by SC-CO_2_ processing of vegetable matter. J. Supercrit. Fluids.

[26] Sarno M., Baldino L., Scudieri C., Cardea S., Ciambelli P., Reverchon E. (2016). Supercritical CO_2_ processing to improve the electrochemical properties of graphene oxide. J. Supercrit. Fluids.

[27] Beckman E., Porter R.S. (1987). Crystallization of bisphenol a polycarbonate induced by supercritical carbon dioxide. J. Polym. Sci. Part B Polym. Phys..

[28] Li B., Zhu X., Hu G., Liu T., Cao G., Zhao L., Yuan W. (2008). Supercritical carbon dioxide-induced melting temperature depression and crystallization of syndiotactic polypropylene. Polym. Eng. Sci..

[29] Zheng H., Zhang J., Du B., Wei Q., Zheng L. (2015). An investigation for the performance of meta-aramid fiber blends treated in supercritical carbon dioxide fluid. Fibers Polym..

[30] Zhang Z., Handa Y.P. (1997). CO_2_-Assisted Melting of Semicrystalline Polymers†. Macromolecules.

[31] Handa Y.P., Kruus P., O’Neill M. (1996). High-pressure calorimetric study of plasticization of poly(methyl methacrylate) by methane, ethylene, and carbon dioxide. J. Polym. Sci. Part B Polym. Phys..

[32] Zhu C., Liu X., Yu X., Zhao N., Liu J., Xu J. (2012). A small-angle X-ray scattering study and molecular dynamics simulation of microvoid evolution during the tensile deformation of carbon fibers. Carbon.

[33] Shioya M., Kawazoe T., Okazaki R., Suei T., Sakurai S., Yamamoto K., Kikutani T. (2008). Small-Angle X-ray Scattering Study on the Tensile Fracture Process of Poly(ethylene terephthalate) Fiber. Macromolecules.

[34] Thünemann A.F., Ruland W. (2000). Microvoids in Polyacrylonitrile Fibers: A Small-Angle X-ray Scattering Study. Macromolecules.

[35] Effler L.J., Fellers J.F. (1992). Structural orientation functions for anisotropic small-angle scattering. J. Phys. D Appl. Phys..

[36] Shioya M., Takaku A. (1985). Characterization of microvoids in carbon fibers by absolute small-angle x-ray measurements on a fiber bundle. J. Appl. Phys..

